# Svapnia, or Purified Opium

**Published:** 1869-02

**Authors:** 


### Svapnia, or Purified Opium.

This is a paper which was contributed to the *Detroit Review of Medicine and Pharmacy*, and discusses the relative effects of narceine, morphine, and codeine. He says morphine acts principally upon the brain; codeine especially on the cerebellum, medulla oblongata, and pneumogastric nerves. When one grain of morphine was injected into one dog, and one grain of codeine into another, both slept calmly for three or four hours. Then the morphine dog waked up wildly, recognized no one, looked haggard, and did not recover his good humor till the next day. The codeine dog waked up bright and playful. The experiment was then reversed, each dog being injected with the other's medicine, with exactly the same results. Morphine produces headache and vomiting very soon, and before a dangerous dose is arrived at, while codeine does not produce these unpleasant effects, and hence requires more care in its exhibition than morphine when given alone. Narceine, as an anodyne and narcotic, may be always employed in place of morphine, and is, in every respect, equal to it in value, and even, in a great many cases, to be preferred to it. In *svapnia*, all the narceine of the opium is retained, but the attempt to isolate it from the codeine and morphine produces a decomposition that cannot be artificially re-arranged, without much expense, and a consequent loss of anodyne and hypnotic power. Hence the peculiar adaptability of this new preparation to all the cases where the calmative alkaloids are applicable. In comparing the relative value of Dr. Squibbs' liquor opii compositus and svapnia, he gives svapnia the preference, because it diffuses its sedative influence over the whole nervous system, instead of being concentrated on the brain. He considers that svapnia is just the medicine required by the profession, which, having all the good effects of opium, does away with many objections to its use, being, in fact, more convenient for every form of exhibition. One grain of svapnia is equivalent to half a grain of extract, or one-third of a grain of morphia.—*Dominion Medical Journal.*

